# A novel soft tissue prediction methodology for orthognathic surgery based on probabilistic finite element modelling

**DOI:** 10.1371/journal.pone.0197209

**Published:** 2018-05-09

**Authors:** Paul G. M. Knoops, Alessandro Borghi, Federica Ruggiero, Giovanni Badiali, Alberto Bianchi, Claudio Marchetti, Naiara Rodriguez-Florez, Richard W. F. Breakey, Owase Jeelani, David J. Dunaway, Silvia Schievano

**Affiliations:** 1 UCL Great Ormond Street Institute of Child Health, London, United Kingdom; 2 Craniofacial Unit, Great Ormond Street Hospital for Children, London, United Kingdom; 3 Oral and Maxillofacial Surgery Unit, St Orsola-Malpighi University Hospital, Bologna, Italy; 4 Department of Biomedical Engineering, Mondragon University, Mondragón, Spain; University of Zaragoza, SPAIN

## Abstract

Repositioning of the maxilla in orthognathic surgery is carried out for functional and aesthetic purposes. Pre-surgical planning tools can predict 3D facial appearance by computing the response of the soft tissue to the changes to the underlying skeleton. The clinical use of commercial prediction software remains controversial, likely due to the deterministic nature of these computational predictions. A novel probabilistic finite element model (FEM) for the prediction of postoperative facial soft tissues is proposed in this paper. A probabilistic FEM was developed and validated on a cohort of eight patients who underwent maxillary repositioning and had pre- and postoperative cone beam computed tomography (CBCT) scans taken. Firstly, a variables correlation assessed various modelling parameters. Secondly, a design of experiments (DOE) provided a range of potential outcomes based on uniformly distributed input parameters, followed by an optimisation. Lastly, the second DOE iteration provided optimised predictions with a probability range. A range of 3D predictions was obtained using the probabilistic FEM and validated using reconstructed soft tissue surfaces from the postoperative CBCT data. The predictions in the nose and upper lip areas accurately include the true postoperative position, whereas the prediction under-estimates the position of the cheeks and lower lip. A probabilistic FEM has been developed and validated for the prediction of the facial appearance following orthognathic surgery. This method shows how inaccuracies in the modelling and uncertainties in executing surgical planning influence the soft tissue prediction and it provides a range of predictions including a minimum and maximum, which may be helpful for patients in understanding the impact of surgery on the face.

## Introduction

Repositioning of the maxilla in orthognathic surgery is carried out for functional and/or aesthetic purposes to correct midface deformities. The most common procedure consists of a bony cut (osteotomy) made superiorly to the teeth, known as a Le Fort I osteotomy, followed by repositioning of the mobilised bone and fixing using metal plates and screws. This operation can be carried out in conjunction with mandibular surgery through bilateral sagittal split osteotomies (BSSO) to improve dental occlusion while achieving satisfactory facial appearance [1].

Pre-surgical planning for these interventions is based predominantly on skull radiographs and, in more specialised centres, also on 3D imaging and computer software tools. These can predict 3D facial appearance by computing the response of the soft tissue to the changes and advancement of the underlying skeleton during maxillofacial and orthognathic surgery, also referred to as ‘soft tissue prediction’ [2–4]. The most common prediction programs are based on mass-spring-models (MSM), mass-tensor-models (MTM) and finite element models (FEM) [5,6]. The first two methods discretise objects into masses and springs, with relatively little computational time required to simulate the surgery, thus making them suitable for clinical applications; disadvantages include the lack of a biomechanical basis–spring constants have no direct correlation to material properties–and volume conservation of the soft tissues cannot be modelled. FEM also rely on discretised objects; however, they represent mathematically defined subdivisions of a continuum problem [5,7,8], where properties can be assigned to each element. These characteristics make them biomechanically relevant and accurate but at substantial computational costs.

Most of the FEM described in literature employ highly detailed anatomical models from magnetic resonance (MR) and computed tomography (CT) imaging in order to improve the accuracy of predicting the soft tissue movements [9]. However, their use is limited to research case studies, whereas most of the MSM and MTM, due to their real-time processing time, have made their way into commercial software such as Surgicase CMF (Materialise, Leuven, Belgium), Dolphin 3D (Dolphin Imaging & Management Solutions, Chatsworth, CA, USA), Simplant O&O (Dentsply-Sirona, York, PA, USA), and 3dMDvultus (3dMD, Atlanta, USA). Despite advances in computational modelling and computer power, clinical use of this software remains controversial and no consensus has been reached in the craniomaxillofacial community [9–17], likely due to the deterministic nature of these computational predictions. Assumptions and simplifications, including the material properties and the mismatch between preoperative planning and actual surgical outcomes in terms of location of osteotomies and amount of repositioning, affect the soft tissue prediction accuracy.

In this study, we propose a probabilistic–instead of a deterministic–FEM approach for soft tissue prediction in orthognathic surgery. Probabilistic FEM have been already adopted in many fields of biomedical engineering [18,19], including craniofacial modelling [20]. Probabilistic in this context describes the inaccuracies that may be present in modelling parameters, as opposed to a deterministic approach where values are assumed without inaccuracy or variation. Examples of probabilistic variables are the material properties which cannot be accurately determined due to measurement inaccuracies or variation within the population analysed. In the FEM these inaccuracies can be modelled by assuming each input variable has a probability density function [20,21]. Firstly, these input parameters are randomised according to a certain distribution (uniform, Gaussain, Weibull, etc.) and a sampling logarithm (Monte Carlo, Latin Hypercube, etc.). Then, multiple deterministic FEM are solved, and after postprocessing, probabilistic distributions for output parameters, e.g. displacement, are obtained [22].

Berthaume et al (2012) [20] have investigated effects of uncertainty and variability of material properties on stress and strain in a primate skull model, showing that including high non-homogeneity, anisotropy, and material property randomness gives large variability in strains and low variability in stresses. Soft tissues and facial appearance, however, were not assessed. We tested and validated our FEM on patients retrospectively using preoperative and postoperative patient data, respectively, as well as surgical planning data.

## Methods

### Patient population and finite element modelling

Eight consecutive patients (mean age 24 years, range 17–36 years, 3f/5m, Table 1) who underwent orthognathic surgery by means of Le Fort I osteotomy maxillary repositioning at St Orsola-Malpighi University Hospital between October 2012 and July 2013 were retrospectively included in this study. All patients had preoperative and postoperative cone beam computed tomography (CBCT) scans. All patients provided written consent and the study was approved by the Independent Ethical Committee of the Sant’Orsola Malpighi University Hospital, Bologna(349/2017/O/OssN). Cutting guides and surgical navigation (eNlite navigation system, Stryker, Freiburg, Germany) were employed to deliver the surgery [23,24].

**Table 1:** Patient details for eight patients who consecutively underwent corrective surgery.

Patient	Age	Gender	Time (days)			Planned maxillary advancement (mm)
			Pre-op CBCT–Surgery	Surgery–Post-op CBCT	Pre-op CBCT–Post-op CBCT	
**P1**	17	M	56	101	157	5
**P2**	19	F	12	42	54	4.5
**P3**	20	M	24	80	104	3.5
**P4**	27	M	26	43	69	4.5
**P5**	18	M	18	94	112	5.5
**P6**	35	F	26	59	85	4.5
**P7**	17	M	13	64	77	5.5
**P8**	32	F	580	49	629	4
**Mean ± SD**	**24 ± 7**		**94 ± 197**	**67 ± 23**	**161 ± 192**	**4.6 ± 0.7**

**Fig 1:**
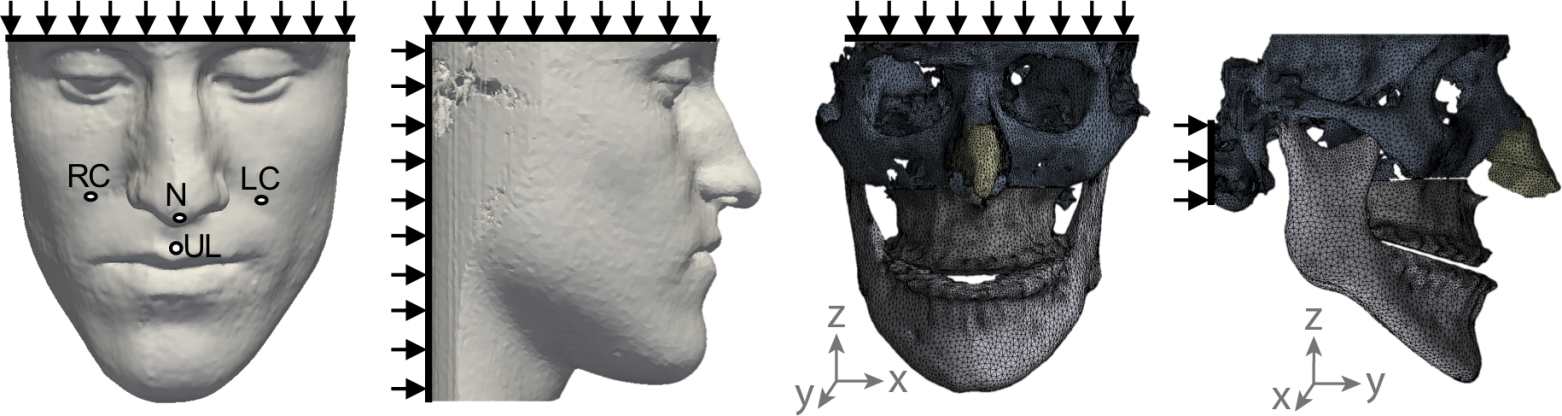
Preoperative CBCT reconstruction of the soft tissues and bones as well as boundary conditions. Boundary conditions are indicated by black arrows and the reference axis by the grey arrows. The soft tissue nodes evaluated in the design of experiments are also indicated: nose (N), upper lip (UL), lower lip (LL), right cheek (RC), and left cheek (LC).

Pre- and postoperative CBCT scans were imported and segmented in Simpleware ScanIP (Synopsis, Mountain View, USA) to reconstruct each patient maxilla, mandible, skull base, nasal cartilage, and soft tissue (Fig 1). Tetrahedral meshes were created using Simpleware ScanIP with a coarseness value of -30, which corresponded to an average for all patients of 233,880 nodes (0.159 nodes/mm^3^) and 1,149,720 elements (0.779 elements/mm^3^). These settings were chosen as an optimal trade-off between accuracy, i.e. stress and strain convergence, and CPU time. Average CPU time for P1 was 705 s per simulation. A complete design of experiments computation with five input variables requires 27 simulations and thus, for P1, corresponds to 19035 s = 5h 17m.

Subsequently, the meshes were imported into Ansys (v17.2, Ansys Inc, Canonsburg, USA). Simulations were set up to replicate the Le Fort I maxilla repositioning procedure, based on the preoperative surgical planning. Nodes were fixed in the following planes (Fig 1): soft tissue posteriorly (y_min_), superiorly (z_max_), and inferiorly (z_min_); skull bone posteriorly (y_min_) and superiorly (z_max_). Furthermore, the mandible was fixed anteriorly-posteriorly (y-axis) and superiorly-inferiorly (z-axis) but set free laterally-medially (x-axis). Advancement of the Le Fort I segment was achieved by anterior translation (y-axis) prescribed on all surface nodes of the osteotomised bone segment. Material properties were set up as describe in the next paragraph.

### Probabilistic finite element analysis

**Fig 2:**
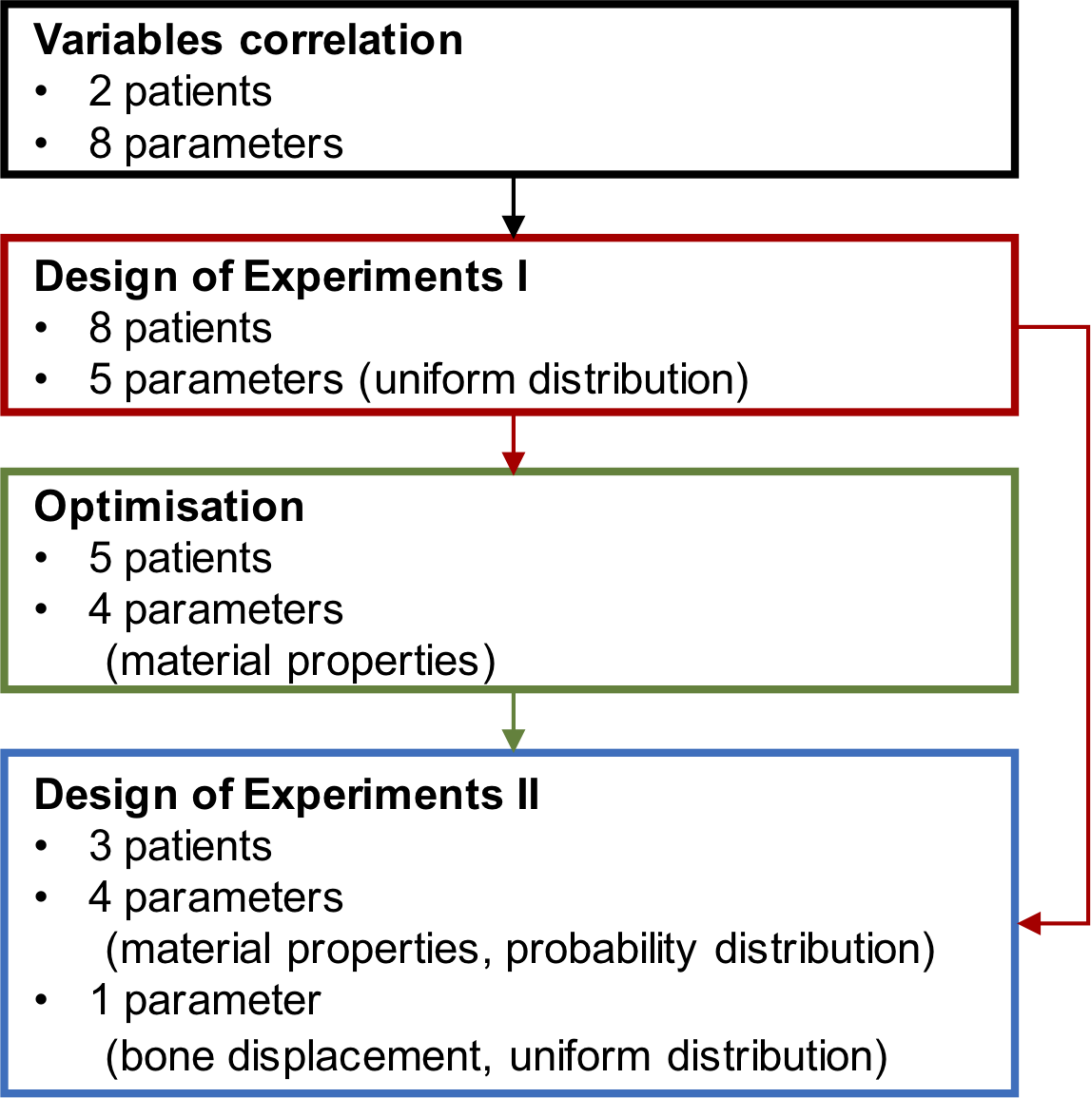
Flowchart of the methodology for the probabilistic finite element analysis. Colour coding: variable correlation, design of experiments I, optimisation on material properties, and design of experiments II.

A correlation analysis of potential input variables was initially performed. Probabilistic FEM was then carried out with a design of experiments (DOE) approach, containing a range of inputs for variables that were found to be significant in the variable correlation analysis. Finally, using the postoperative CBCT, the material properties were optimised in a subgroup of patients and the second iteration of DOE was carried out using those optimised values. The optimisation process sought to match the soft tissue predictions from the probabilistic FEM to the soft tissue location in the postoperative CBCT and, thereby, reduce the corresponding material property input ranges. A flowchart is shown in Fig 2. Variable correlation, DOE, and optimisation were all carried out in Ansys [22].

#### Variable correlation

A correlation analysis was carried out to assess which input variables have a significant effect on output variables in the probabilistic model. This correlation was performed in P1 and validated in P2. Significant variables were then implemented initially with a uniform range, non-significant variables were implemented using the mean value of the range. Input variables considered were: *E*_bone_ and *ν*_bone_; *E*_cart_ and *ν*_cart_; *E*_soft_, *ν*_soft_ and *G*_soft_; and *x*_bone_, where *E* describes Young’s Modulus, *ν* Poisson’s ratio, *G* viscoelasticity, and *x* bone advancement. Material property ranges were based on literature [25–31] (Table 2). The soft tissue was assumed to be incompressible and, therefore, no volumetric relaxation was implemented. Normalised shear relaxation, however, was implemented using Prony series with values from literature [30,31]:

$$G(t)=1-\sum_{1}^{n}g_{i}(1-e^{-t/\tau_{i}})$$

where *G(t)* describes normalised shear relaxation over time, *g*_i_ the relaxation and *τ*_i_ the characteristic relaxation constant for *n* components, and *t* time. Shear relaxation was implemented with two terms [30], representing a total of 62.5% relaxation, see equation (2). Subsequently, a scaling factor (*G*_soft_) ranging between 0.488 and 1.512 was applied to represent a minimum of 30.5% and a maximum of 94.4% relaxation. The relaxation constants were assumed constant in the DOE experiment.

$$g_{i}=\left[\begin{array}{c} 0.325\\ 0.3 \end{array}\right],\tau_{i}=\left[\begin{array}{c} 1\\ 6 \end{array}\right]$$

**Table 2:** Input parameters, and minimum and maximum values of the material properties.

Input parameter	Min	Max
**Bone [25,26]**		
***E* (MPa)**	5000	15000
***ν* (-)**	0.2	0.4
**Cartilage [27–29]**		
***E* (MPa)**	0.5	5
***ν* (-)**	0.26	0.38
**Soft tissue [30,31]**		
***E* (MPa)**	0.1	1
***ν* (-)**	0.45	0.499
***G* (%)**	30.5	94.4

*E*: Young’s modulus, *ν*: Poisson’s ratio, G: viscoelasticity (Prony series), *x*: advancement, *soft*: soft tissue, *cart*: cartilage.

The ability of the surgeons to reproduce the preoperative planning in terms of osteotomy location and maxillary advancement vector was also considered. The osteotomy location in the FEM was derived from the surgical planning and assumed to be highly accurate because cutting guides and surgical navigation were used during surgery [23]. The bone advancement vector typically is determined preoperatively (Table 1), however, the postoperative bone position differs from the intended position throughout the bone segment as much as ±2 mm [17,23]. Therefore, the uncertainty on the bone advancement vector was set at ±2 mm.

The outputs for correlation were the soft tissue prediction in five points selected as representative of the various areas of the face likely to be affected by orthognathic surgery (Fig 1): the nose tip (N, cephalometric equivalent: pronasale), upper lip (UL, labrale superius), lower lip (LL, labrale inferius), and right (RC) and left cheek (LC). RC and LC are defined laterally to the nose tip and inferiorly to the centre of the eye, defined as the middle between both canthi.

Spearman correlation with significance at 95% was set as inclusion criteria for the variables in the DOE approach.

#### Design of experiments I

DOE was used to investigate further the relationship between those variables that satisfied the correlation threshold and the clinical outputs. The following steps were carried out for all eight patients. An optimal space-filling (OSF) algorithm was used for input parameter generation. OSF is an extended Latin Hypercube Sampling (LHS) method which ensures uniform parameter distribution amongst the set range while minimising the number of simulations [22]. For each patient, 27 runs were required to compute the DOE matrix. Response surfaces based on genetic aggregation, depicting the input-output relationships, were generated from the DOE as well as sensitivity curves. Soft tissue prediction ranges were compared to the postoperative CBCT scans in the five points described above: N, UL, LL, RC, and LC.

#### Optimisation of material properties

Out of the eight patients, five were randomly selected for optimisation (P2, P3, P5, P7, and P8) of the input material property variables and three for the second iteration of DOE and subsequent validation (P1, P4 and P6) of the soft tissue displacement predictions.

For all soft tissue points but LL, the optimisation algorithm attempts to find the exact solution as determined by CBCT, if the value lies within the predicted range, otherwise, it seeks to minimise or maximise the soft tissue point. The lower lip is omitted from optimisation since the BSSO were not included in the modelling.

The optimisation problem is nontrivial, as due to conflicting optimisation goals no unique solution exists that satisfies all optimisation objectives [32–34]. Therefore, a multi-objective optimisation method was adopted that seeks Pareto optimal solutions. An *a priori*, goal driven multi-objective genetic algorithm (MOGA) was selected, with equally weighted objectives as listed in the above paragraph. Within the MOGA, based on the Ansys default settings, 1000 synthetic solutions were generated per iteration based on the response surfaces, with a maximum of 10 iterations. Convergence stability was set at 2% with a Pareto criterion of 70%. The best 100 synthetic simulations were extracted, representing an optimised subset within the bounds of the original DOE results and thus corresponding to improved soft tissue prediction and reduced material property range.

#### Design of experiments II

The 100 candidate points (*m* = 100) for each of the material property inputs (*n* = 4 from the correlation analysis as discussed in the Results) represent an *m x n* optimised input matrix. Row-wise superimposition of these matrices for P2, P3, P5, P7, and P8 provides the training matrices. Four Weibull distributions were fitted using Matlab (v2016b, MathWorks, Natick, USA) for each vector of length *5m*. These population-specific material property variable distributions were subsequently used as inputs for the second iteration of DOE and were tested on P1, P4, and P6. The key difference with the first DOE iteration is that each material property has a probability distribution based on patient data as opposed to a uniform range based on minima and maxima from literature.

## Results

The variable correlation analysis showed that only five out of the eight input parameters were significantly correlated (p<0.05) to at least one of the five output parameters: *E*_cart_, *E*_soft_, *ν*_soft_, *G*_soft,_ and *x*_bone_. Table 3 shows the correlation matrix for P1; the same trends were found for P2. Furthermore, Table 3 shows that bone advancement is most strongly correlated to all five soft tissue points (0.60 ≤ r ≤ 0.98) followed by soft tissue material properties (r ≤ 0.50) and cartilage material properties (r ≤ 0.32).

**Table 3:** Variable correlation results for patient P1 between various input variables and output.

Input	Output				
	N	UL	LL	RC	LC
***E***_**soft**_	r = 0.50*** CI = 0.33, 0.64	r = -0.03	r = -0.31** CI = -0.49, -0.11	r = 0.07	r = 0.09
***ν***_**soft**_	r = 0.24* CI = 0.03, 0.43	r = 0.18	r = 0.03	r = 0.16	r = 0.03
***G***_**soft**_	r = -0.30** CI = -0.48, -0.10	r = 0.21* CI = 0.00, 0.40	r = 0.37*** CI = 0.18, 0.54	r = 0.08	r = -0.05
***E***_**bone**_	r = -0.13	r = 0.01	r = 0.06	r = -0.07	r = 0.07
***ν***_**bone**_	r = -0.01	r = -0.03	r = -0.04	r = -0.01	r = 0.00
***E***_**cart**_	r = -0.32*** CI = -0.49, -0.12	r = 0.08	r = 0.30** CI = 0.10, 0.48	r = 0.09	r = 0.06
***ν***_**cart**_	r = 0.02	r = 0.05	r = -0.07	r = -0.04	r = -0.03
***x***_**bone**_	r = 0.60*** CI = 0.45, 0.72	r = 0.98*** CI = 0.97, 0.98	r = 0.79*** CI = 0.08, 0.47	r = 0.98*** CI = 0.97, 0.99	r = 0.97*** CI = 0.96, 0.98

*E*: Young’s modulus, *ν*: Poisson’s ratio, G: viscoelasticity (Prony series), *x*: advancement, *soft*: soft tissue, *cart*: cartilage, N: nose, UL: upper lip, LL: lower lip, RC: right cheek, LC: left cheek, r: Pearson’s correlation, CI: confidence interval

***: p<0.001

**: p<0.01

*: p<0.05.

**Fig 3:**
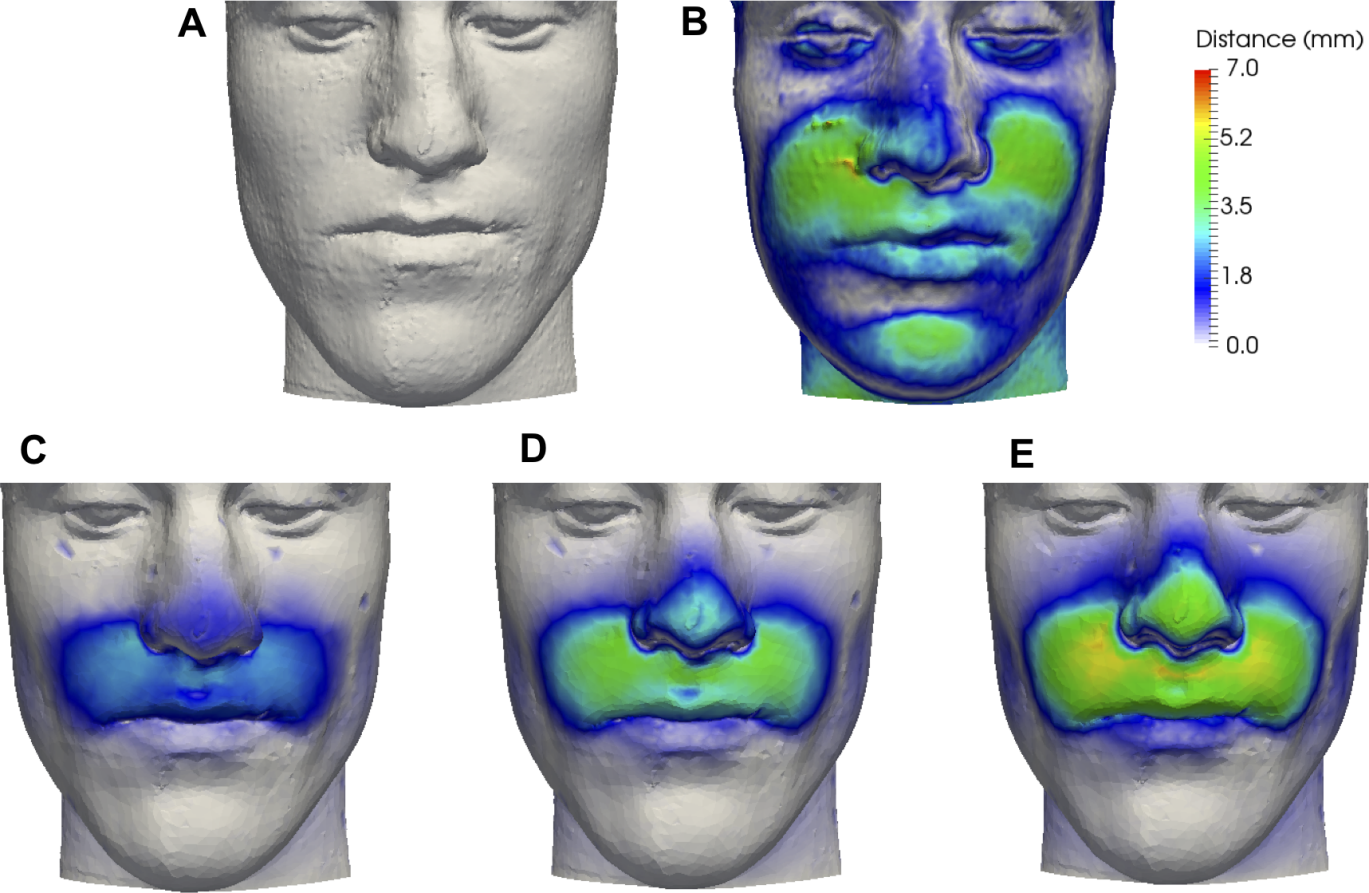
Patient 1 soft tissue prediction minimum and maximum. (A) Overall minimum soft tissue prediction displacements with *G*_*soft*_ = 94.4%, *E*_*soft*_ = 0.1 MPa, *ν*_*soft*_ = 0.45, *E*_*cart*_ = 0.5 MPa, *x*_*disp*_ = 3 mm, and (B) maximum soft tissue prediction displacements with *G*_*soft*_ = 30.5%, *E*_*soft*_ = 1 MPa, *ν*_*soft*_ = 0.49, *E*_*cart*_ = 5 MPa, *x*_*disp*_ = 7 mm. Blue depicts no change from the postoperative CBCT (0 mm), bright red depicts a maximum change (6 mm).

**Fig 4:**
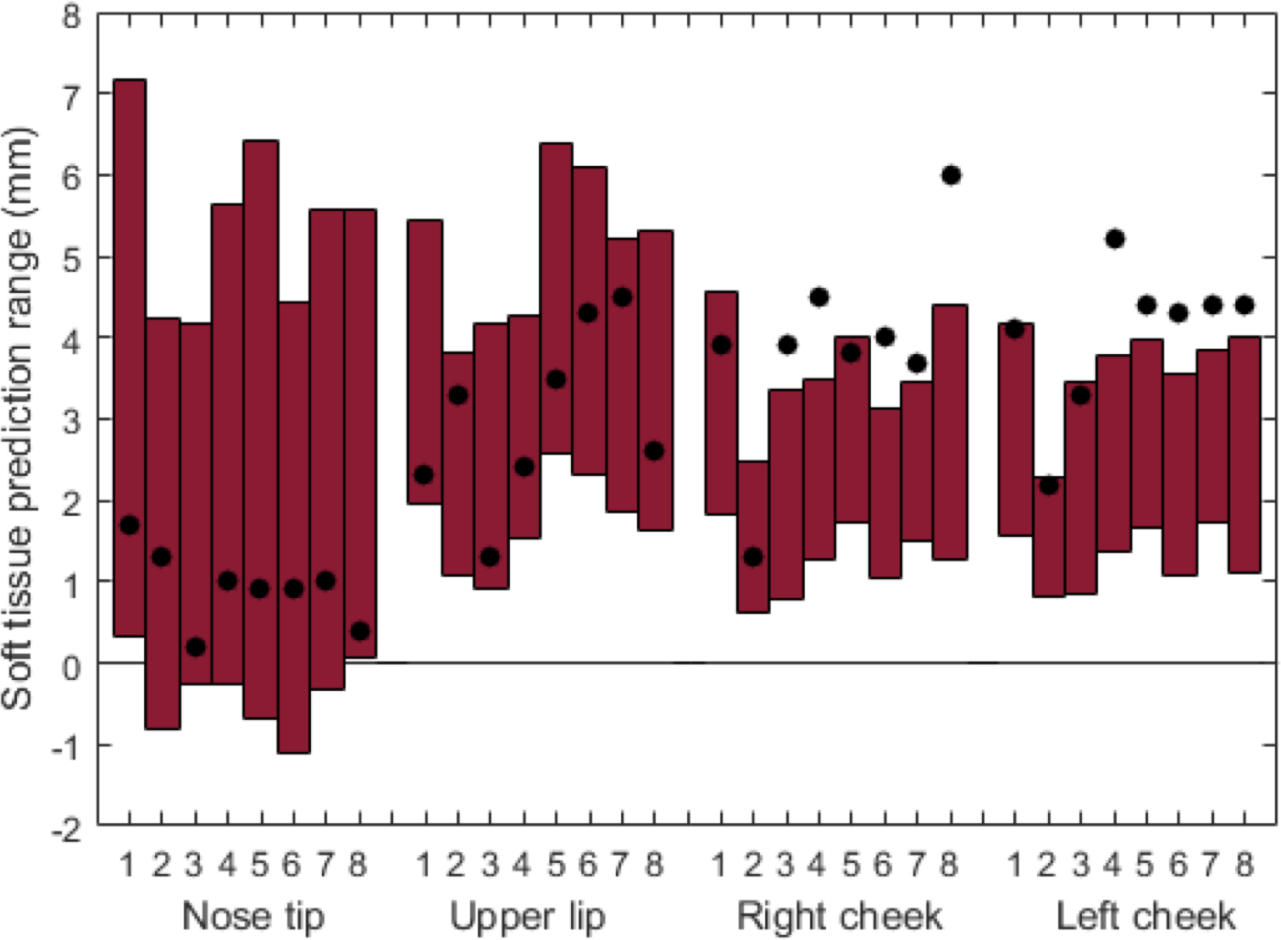
Design of experiments I: Soft tissue soft prediction ranges in five points for all patients. Points include nose tip, upper lip, lower lip, right cheek, and left cheek. The vertical bars depict the range of soft tissue prediction; the black dots represent the soft tissue displacement from the postoperative CBCT. The baseline (0 mm) is the preoperative CBCT.

DOE I was carried out for all eight patients with a uniform distribution range for the five significant input variables and a mean value for the non-significant input variables. Fig 3 shows an example of the prediction displacements from preoperative CBCT for P1, based on the overall minimum and maximum soft tissue displacements. Fig 4 shows the displacement ranges predicted by the probabilistic FEM (red bars) in each soft tissue output point, alongside the postoperative CBCT (black dots) actual displacements. The baseline value (0 mm) represents the preoperative CBCT. The displacement predictions in N and UL were including the actual CBCT displacements for all patients, while for LC and RC in most patients, the displacement predictions were underestimating the real advancement.

**Fig 5:**
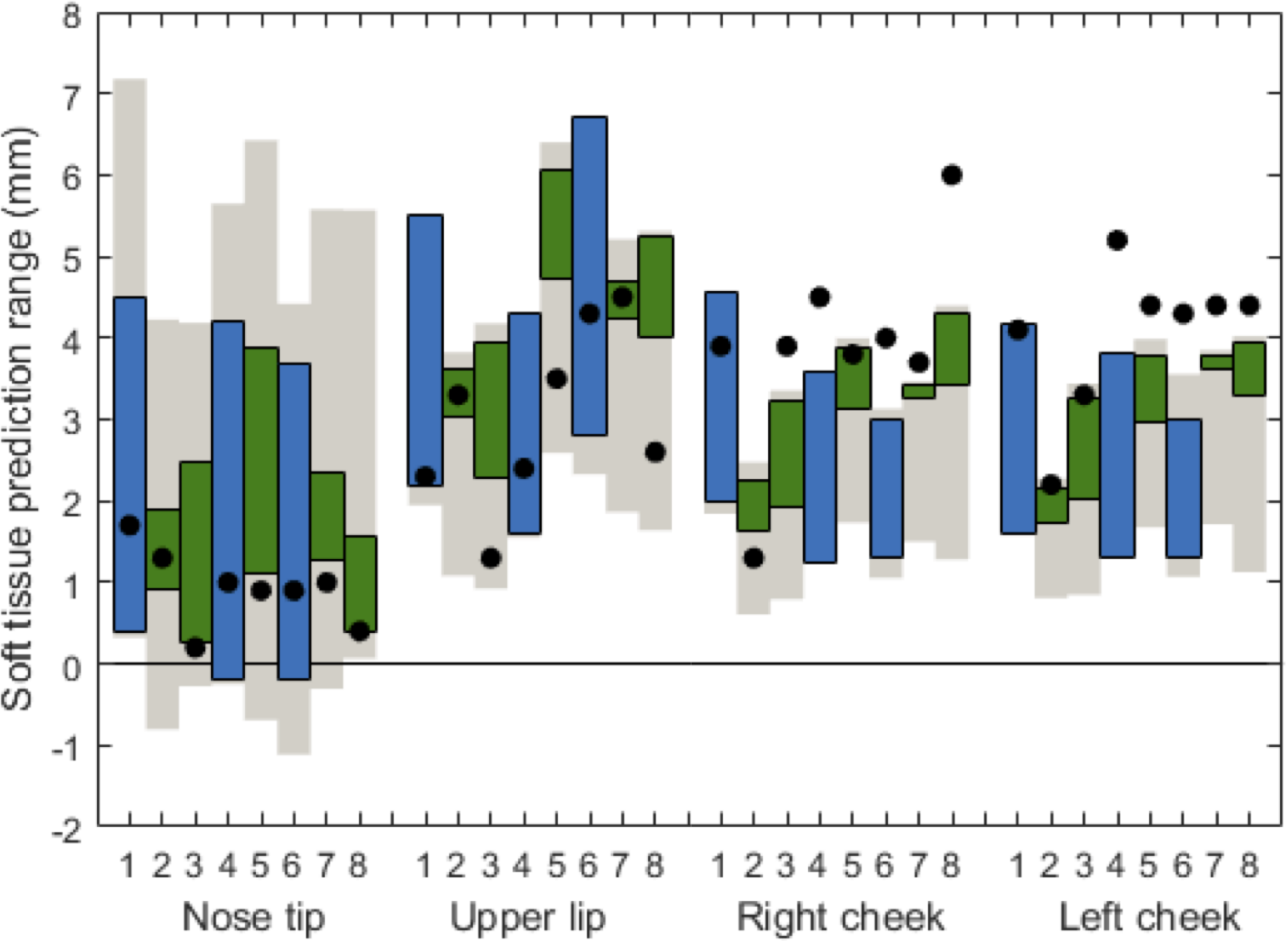
Soft prediction ranges from design of experiments I (green) and design of experiments II (blue) following optimisation. Green (P2, P3, P5, P7, and P8): based on design of experiments I and subsequent optimisation on postoperative CBCT scans. Blue (P1, P4, and P6): soft tissue ranges from design of experiments II based on optimised material properties.

The MOGA optimisation produced 100 synthetic simulations per patient that provide subsets of the predictions for P2, P3, P5, P7, and P8, as shown in Fig 5 with green bars. The ranges were optimised for four points (N, UL, RC, and LC), while also negatively affecting the prediction range in point LL.

**Fig 6:**
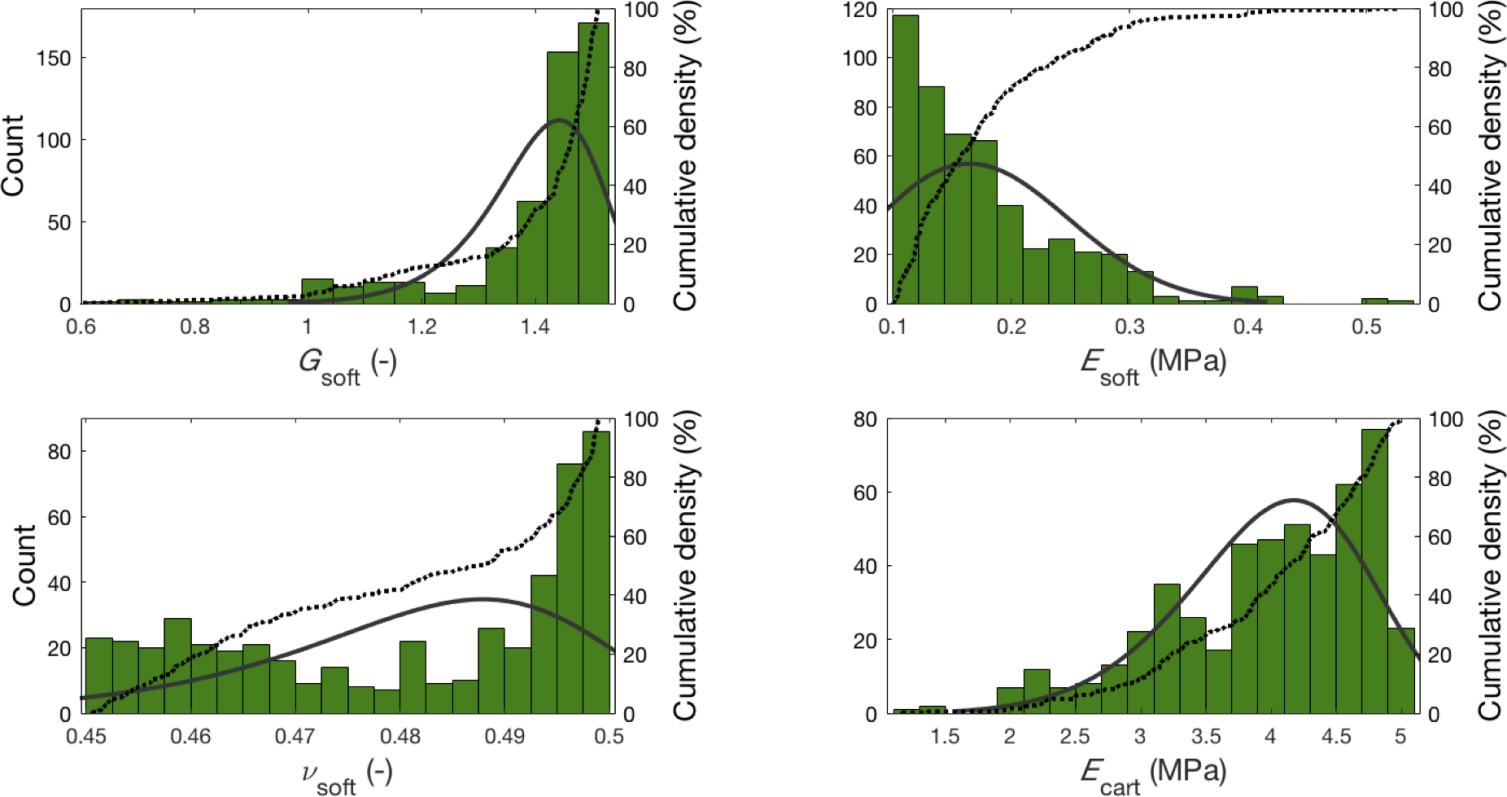
Histogram depicting the distribution of material properties following optimisation. Green bars display all training data from P2, P3, P5, P7 and P8, as well as a fitted Weibull curve (solid black line) and cumulative density (dotted black line). *G*: viscoelastic scale factor, *E*: Young’s Modulus, *ν*: Poisson’s ratio, *soft*: soft tissue, *cart*: cartilage.

The corresponding material property data for the 100 synthetic simulations were summed for those patients and a Weibull curve was fitted to these superimposed datasets, as shown in Fig 6.

The second DOE iteration for the three remaining patients (P1, P4, and P6), using the fitted Weibull curve representing the distribution of material properties from clinical patient data, resulted in the prediction ranges as shown in Fig 5 with blue bars. These optimised predictions for N and UL were including the actual CBCT displacements whereas in RC and LC the predictions were underestimating, similar to the results obtained with DOE I but with a refined prediction range.

**Fig 7:**
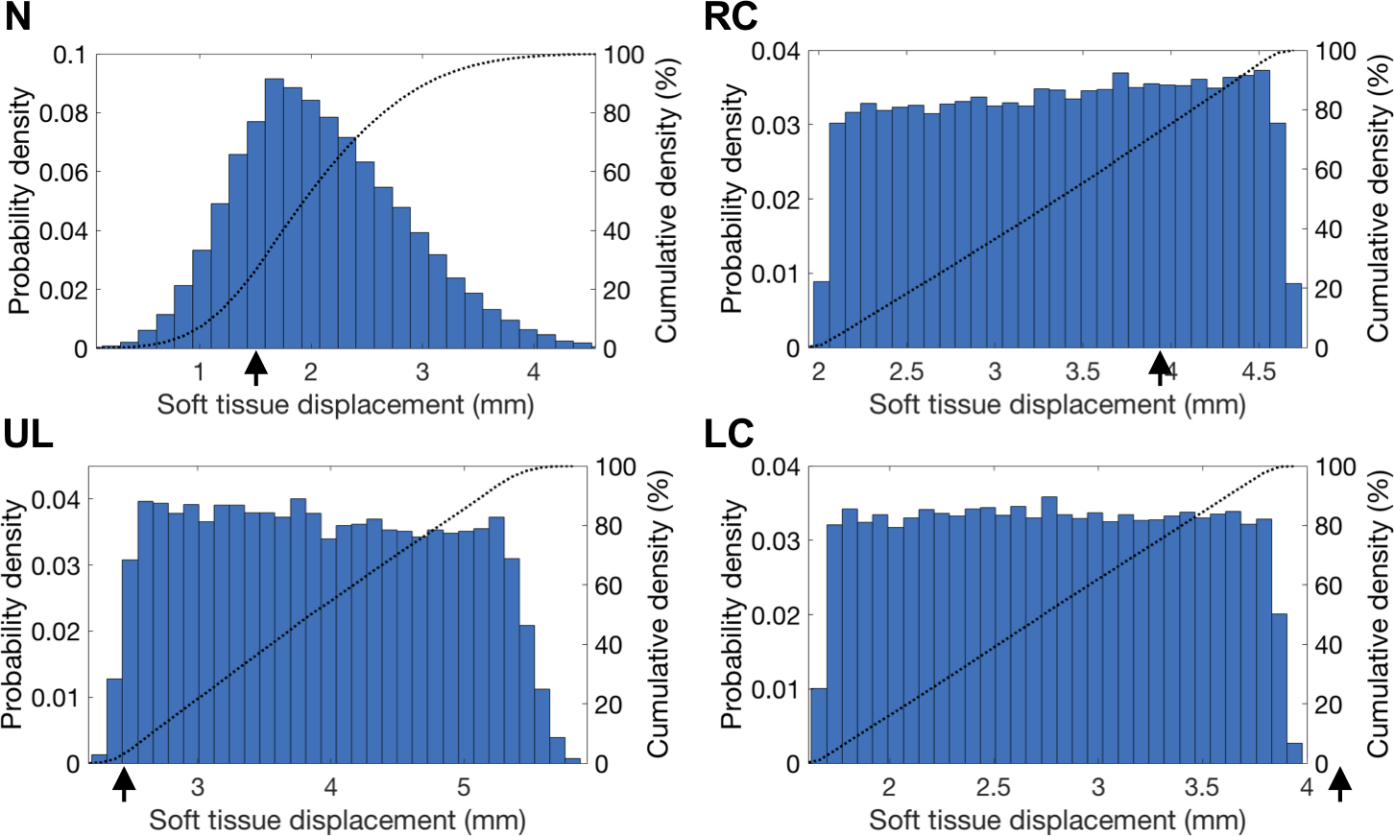
Patient 1: Soft tissue prediction probability histograms. Showing the nose (N), upper lip (UL), lower lip (LL), right cheek (RC) and left cheek (LC), as well as cumulative density plots (dotted lines). The black arrows on the x-axis depict the CBCT postoperative soft tissue position; preoperative position is equal to 0 mm displacement. The histograms for P4 and P6 show similar probability curves.

The probability distribution of the soft tissue predictions in N, UL, LL, RC, and LC is shown in Fig 7 for P1 and is representative for P4 and P6. The blue bars of the histogram show the distribution and the dotted lines indicate the cumulative density. Furthermore, the black arrows indicate the true displacement from the postoperative CBCT.

**Fig 8:**
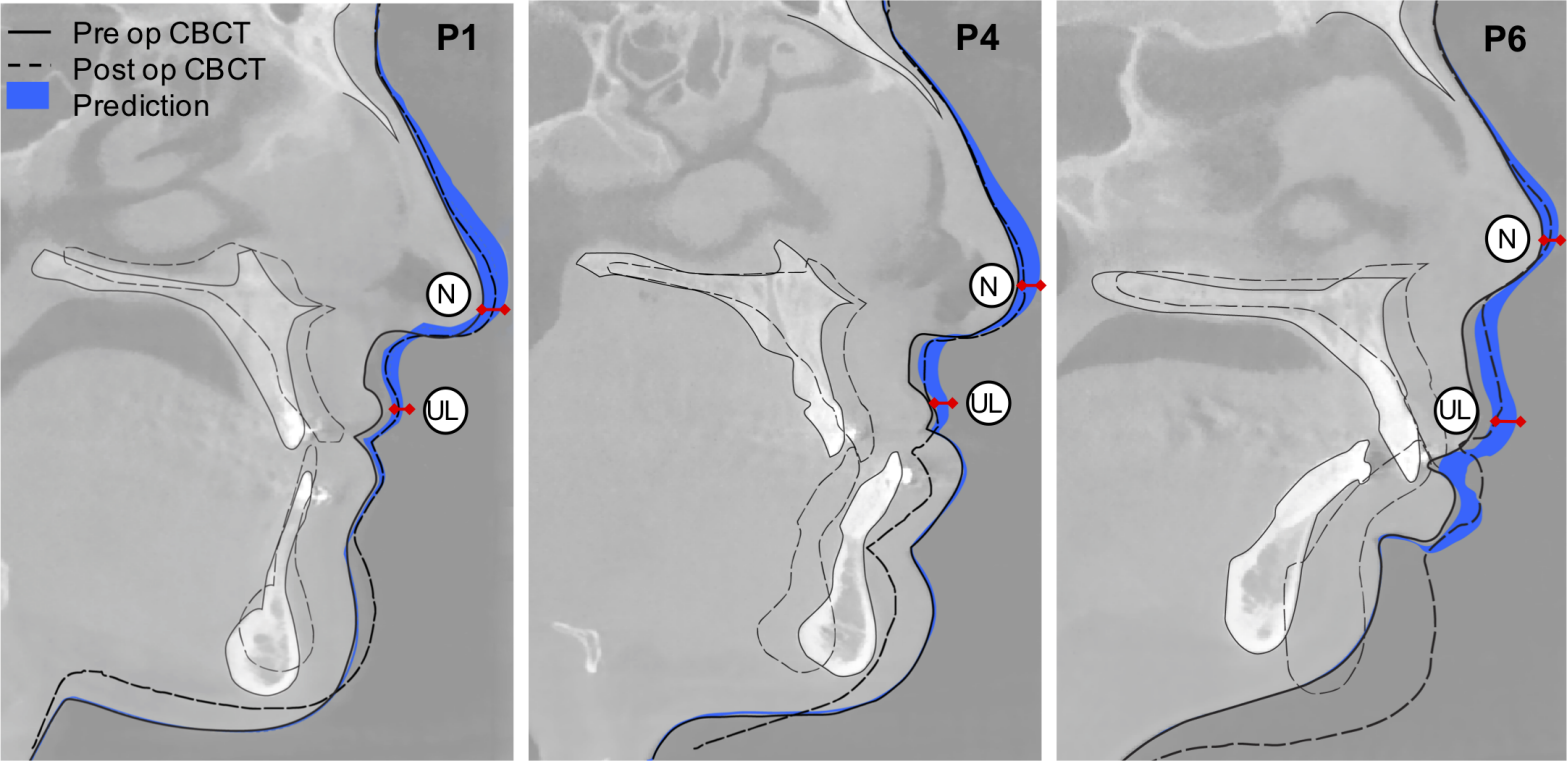
Midline 2D views for P1, P4, and P6. For each patient, preoperative CBCT (solid black line), postoperative CBCT (dashed line), and probabilistic soft tissue prediction (blue area) are shown, as well as indications for the nose (N), upper lip (UL), and lower lip (LL) points.

Fig 8 shows the 2D midline prediction range, following DOE II, alongside the preoperative and postoperative CBCT. The soft tissue prediction range includes the postoperative position in the nose and upper lip areas but deviates from the postoperative CBCT in the chin area as a result of excluding BSSO.

## Discussion

A probabilistic FEM approach has been described in this study for the prediction of soft tissue repositioning after orthognathic surgery. The advantage of the probabilistic FEM is that it considers uncertainties in material properties as well as uncertainties relating to the osteotomy and maxillary repositioning. The resulting soft tissue prediction, therefore, does not consist of a single deterministic outcome, but rather of a range of soft tissue outcomes including a minimum and a maximum. This addresses the inability of current soft tissue prediction software to accurately capture the 3D facial outcome with one single result, thus may be beneficial for patient communication as it can demonstrate the impact of maxillofacial surgery on their face more realistically. Clinical data from eight patients who underwent Le Fort I maxillary repositioning were used to train and validate the model, but this approach may also be applied to other craniofacial interventions such as Le Fort III, monobloc, and bipartition osteotomies [35,36] as well as other types of aesthetic and reconstructive surgery.

The uncertainty in bone position had the largest influence on the predicted soft tissue displacements, followed by the soft tissue material properties. This suggests that the accuracy of any FEM soft tissue prediction is mostly defined by the ability of the surgeon to match the pre-procedural plans for bone repositioning and this reflects the technical difficulties in accurately reproducing pre-operatively planned maxillary movements during surgery. A limitation of our probabilistic FEM is that maxillary advancement is modelled solely in the anterior-posterior plane, without rotation. Additionally, BSSO as well as autorotation of the mandible have not been taken into account.

The approach provides full 3D predictions for the face: five specific landmark points were used for comparison with clinical data along with the 2D facial midline. The postoperative nose tip and upper lip positions were within the predicted range in all patients. The postoperative position of the lower lip heavily depended on the position of the mandible. Since the FEM considered only the bone changes in the maxilla and not those of BSSO, the predicted position of the soft tissues in the jaw area was not considered in the optimisation. The postoperative position of the cheeks was within the predicted soft tissue range in three patients and under-predicted in five patients. This discrepancy may be due to swelling, since the time between surgery and postoperative CBCT was 67 ± 23 days and swelling in orthognathic surgery is reported to reduce by approximately 60% after 1 month and 83% after 3 months [37]. This is one limitation of this retrospective study as the postoperative CT scans were acquired to assess skeletal outcomes prior to orthodontic movements rather than the soft tissue results. Another cause of underestimation might be the lack of titanium bone plates in the model, which have a standard thickness of 1 mm [38].

The accuracy of predictive FEM was increased by employing a detailed anatomy together with appropriate population-specific material properties [9]. Indeed, the presented probabilistic method relied on an initial range of material properties from the literature followed by an optimisation step using training data from the specific population. However, in this FEM, the facial soft tissues were modelled as a homogeneous layer with no subdivision between skin, superficial muscular aponeurotic system (SMAS) and superficial fat. Inhomogeneous tissue models have been described [39] and constitutive model parameters have been reported for skin, SMAS and fat [40,41]. Further work will be undertaken to implement an anatomically detailed mesh with distinct soft tissue layers and thereby improve accuracy and reduce the range between minimum and maximum predictions. Additionally, it can be time-consuming to obtain patient-specific anatomical meshes. Zhang et al (2016) [42] have shown that thin plate spline mesh morphing can be used to warp a template mesh to a patient-specific mesh. Furthermore, the focus of this study was on the probabilistic methodology with simplified behaviour of the materials. Non-linear constitutive equations would better describe the properties of various soft tissue components and it is expected to increase the simulation time as an iterative non-linear solver (Newton-Rapshon method in Ansys) would have to be employed. Accuracy would certainly increase; however, it is unclear if the level of improvement would justify the higher simulation time.

The optimisation process and DOE II relies on training data used to improve the soft tissue prediction. Inherently, the accuracy of the soft tissue prediction model will depend on the training data, which might lead to inaccuracies when interpolating these results onto other cohorts, for example, paediatric patients. Other statistical approaches relying on patient data have been described in the literature [43–45].

Lastly, it must be noted that FEM come at a computational cost and a similar probabilistic approach could be implemented based on MSM or MTM models, thereby improving speed. The pFEM approach allows for great control over the modelling parameters, something that is lacking in commercial software, which might be preferable over speed when looking at specific cases that fall outside the normal population, e.g. severe congenital facial deformities. Furthermore, in a clinical setting, a DOE optimisation might be used firstly to obtain population-specific parameters from a retrospective cohort after which these parameters can be used for a traditional FEM approach for a prospective case.

The ease of implementation using readily available tools as described in this paper was prioritised and speed within FEM will be optimised in further developments. This proof of concept shows the feasibility of using probabilistic FEM for soft tissue prediction.

## Conclusions

A probabilistic FEM has been implemented for the prediction of the facial soft tissues following orthognathic surgery and has been validated on clinical data of eight patients. The advantage of a probabilistic approach over traditional deterministic approaches is that it shows how inaccuracies in the modelling and uncertainties in executing surgical planning can influence the soft tissue prediction. Thereby, it illustrates the impact of maxillofacial surgery on the face within a confidence interval, in 3D, which may be helpful for patients and surgeons. The proposed pipeline is not limited to prediction of soft tissue changes following a Le Fort I osteotomy, but it could be applied to other craniofacial interventions as well as other types of plastic surgery.
